# Supplementary material for: Genome-Wide Identification of the Rose SWEET Gene Family and Their Different Expression Profiles in Cold Response between Two Rose Species
Source: Plants (Basel). 2023 Mar 28;12(7):1474. doi: 10.3390/plants12071474 (PMC10096651; doi:10.3390/plants12071474)
Supplement: Supplementary file 1 [file plants-12-01474-s001.zip › Supplementary file6-Table S5.pdf]

Table S5. The numbers of predicted cis-elements in the promoters of 25 *RcSWEET* genes.

Element	ABRE	ARE	AuxRR-core	Box 4	CGTCA-motif	circadian	ERE	GARE-motif	LTR	MBS	P-box	TATC-box	TCA-element	TC-rich	TGACG-motif	TGA-element	WUN-motif
Annotation	ABA	Anaerobic	Auxin	Light	MeJA	Circadian control	Ethylene	Gibberellin	Low temperature	Drought	Gibberellin	Gibberellin	Salicylic acid	Defense and stress	MeJA	Auxin	Wound
RcSWEET1		1		2	1		1					1			1	2	1
RcSWEET2a	5	4				2	1	1	2	2	1						
RcSWEET2b	4	2		4	1	2			2			1		1	1	2	
RcSWEET3		7		4						2	1		1				
RcSWEET4a	3	3		7		1	3			1		1		1			
RcSWEET4b	3	3			2		1	1	1	1		1		1	2		2
RcSWEET5a	3	3		2	4							1			4	2	
RcSWEET5b		6		2	2	1				1			1		2	1	
RcSWEET5c	2	2		1						1	1		1				
RcSWEET5d	4	3		2	1			1						1	1		
RcSWEET5e	6	2	1	4	1		2						1	1	1	1	1
RcSWEET5f	2	1		2	1	1			1				1	2	1		
RcSWEET5g	6	4			2		1		2	2			1		2	1	
RcSWEET5h		2		1				1	1	3			1			1	
RcSWEET9	2			3	1			1		1					1	2	
RcSWEET10a	1	1		3				1		3			1				1
RcSWEET10b	2	4		3					1	3							
RcSWEET10c	9	5	1	2			1		1				1	1		1	3
RcSWEET11a	4	1			1		3		1		1	1			1		1
RcSWEET11b	5	5		4	1					1			1		1		1
RcSWEET12	2			7	2		1							2	2		2
RcSWEET15a	4	3		6	3	1		1	1				1	1	3		
RcSWEET15b	4			2	3				2		1	2	1		3	1	1
RcSWEET17a	1	1		2	1		1		2		1				1	1	
RcSWEET17b	3	1		5	2				1						2	1	1
