# Supplementary figures and images for: Genome-Wide Identification of the Rose SWEET Gene Family and Their Different Expression Profiles in Cold Response between Two Rose Species
Source: Plants (Basel). 2023 Mar 28;12(7):1474. doi: 10.3390/plants12071474 (PMC10096651; doi:10.3390/plants12071474)

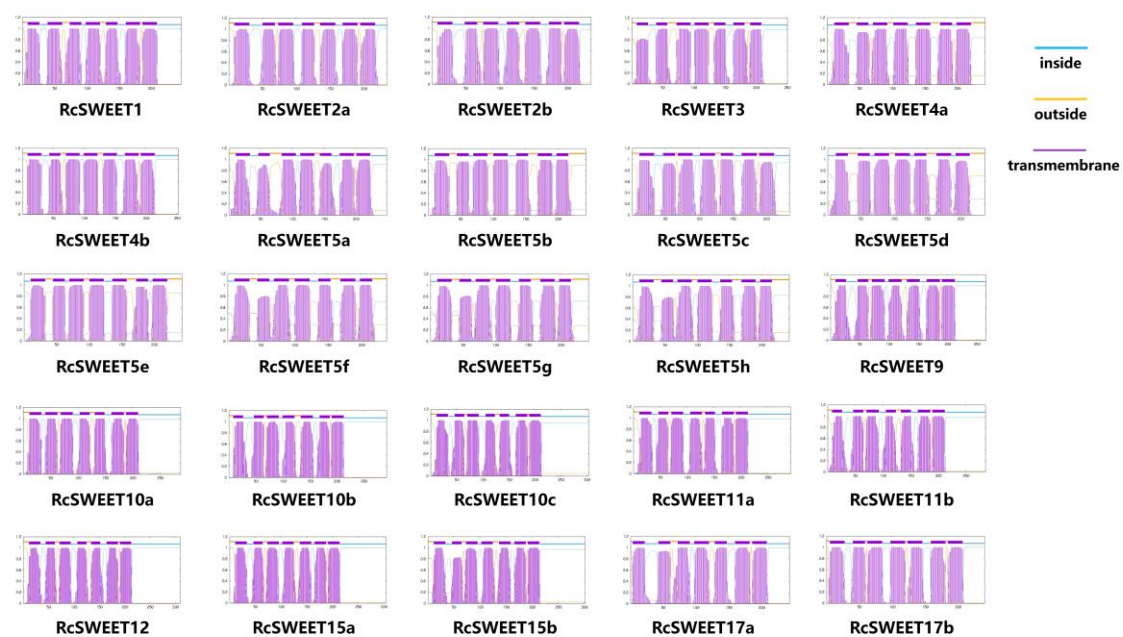

**Figure S1.** The transmembrane structure of 25 RcSWEET proteins.

Supplement: Supplementary file 1 [file plants-12-01474-s001.zip › Supplementary file1-Figure S1.pdf]
